# Endosomal MrGPRX1 signaling sensitizes TRPV1 to enhance itch

**DOI:** 10.3389/fnmol.2026.1880762

**Published:** 2026-07-01

**Authors:** Paz Duran, Jeffri S. Retamal, Marcella de Amorim Ferreira, Kai Trevett, Evan Chen, Dane D. Jensen

**Affiliations:** 1 Department of Molecular Pathobiology, College of Dentistry, New York University, New York, NY, United States; 2 Translational Research Center, College of Dentistry, New York University, New York, NY, United States; 3 NYU Pain Research Center, College of Dentistry, New York University, New York, NY, United States; 4 Department of Chemistry and Biology, University of Santiago Chile, Santiago, Chile

**Keywords:** endosome, intracellular signaling, MrGPRs, signal transduction, transient receptor potential channels (TRP Channels)

## Abstract

G protein-coupled receptors (GPCRs) and TRPV (transient receptor potential vanilloid) channels are crucial for signal transduction in physiological processes, including neurotransmission, pain, and itch. Downstream effectors of GPCR signaling can directly stimulate TRPV channels or enhance their sensitivity to stimuli, a process known as TRPV sensitization. Traditionally, GPCRs are activated at the cell surface by extracellular agonists, triggering signaling cascades. Recent evidence suggests GPCRs continue to signal from intracellular organelles. The human Mas-related G-protein coupled receptor X1 (MrGPRX1) is a GPCR expressed in primary sensory neurons involved in nociception and pruritus. Recent studies demonstrated how intracellular GPCR signaling regulates neuronal activity. However, there is no evidence characterizing MrGPRX1 trafficking or intracellular signaling. Herein, we characterized MrGPRX1 signaling within the endosomal network and its role in sensitizing TRPV1 channels to enhance itch signaling. Utilizing subcellular targeted biosensors, we demonstrated MrGPRX1 can traffic and signal from endosomes. Immunofluorescence analysis showed that MrGPRX1 internalizes following BAM8-22 stimulation. BRET assays revealed that MrGPRX1 activation induces Gα_*q*_ and β-arrestin-1 recruitment to the plasma membrane and early endosomes. Inhibition of dynamin or clathrin blocked BAM8-22-induced MrGPRX1 endocytosis and decreased nuclear extracellular signal-regulated kinase (ERK) signaling. Calcium signaling confirmed that MrGPRX1-mediated TRPV1 sensitization is mediated by protein kinase C and ERK activation. Our findings reveal a novel role for MrGPRX1 endosomal signaling in TRPV1 sensitization. Understanding the mechanisms of MrGPRX1 signaling offers valuable insights into differentiating between pain and itch pathways, aiding in the development of targeted therapies for chronic pain and persistent itch.

## Introduction

Pruritus (itch) is a significant unresolved clinical issue, described as an unpleasant sensation that provokes the desire to scratch. This conserved mammalian response serves as a physiological self-protective mechanism, physically removing foreign objects or irritants from the skin. However, chronic or intense acute itch can lead to great discomfort when not manage properly (Cevikbas and Lerner, 2020). Itch affects one in four adults, severely diminishing the quality of life, increasing stress levels and making itch one of the most debilitating conditions in patient care (Khalil et al., 2024; Roh et al., 2022). Despite its prevalence, there are few therapies to manage chronic itch as the molecular, cellular, and neural circuit mechanisms underlying this condition are not yet fully understood. Thus, it is essential to characterize the molecular and cellular mechanisms that regulate itch in order to develop targeted therapies and improve patient outcomes.

Itch irritants on the skin are detected by peripheral sensory neurons expressing specific receptors and ion channels that respond to diverse thermal, mechanical, and chemical noxious stimuli. Two key classes of itch sensing receptors are G protein-coupled receptors (GPCRs) and transient receptor potential (TRPs) ion channels. GPCRs are essential pruritogen detectors, detecting most itch-inducing molecules controlling both histamine-dependent and -independent itch pathways (Bautista et al., 2014; Liu et al., 2009). Similarly, TRP channels like the transient receptor potential vanilloid 1 (TRPV1) play a large role in regulating itch sensation (Yu et al., 2016). A greater understating of the interplay between GPCRs and TRP channels is necessary as GPCRs are known to modulate TRP channels, altering sensory neuron activity and gating between itch and pain sensation (Chen et al., 2016; Peng et al., 2020; Poole et al., 2013; Retamal et al., 2021; Zhao et al., 2014).

TRPs are a group of non-selective cation channels expressed in nociceptors that allow ions to pass through the plasma membrane (Rosenbaum Emir, 2017). These channels are crucial for detecting and responding to noxious stimuli; therefore, they play critical roles in pain, inflammation, and itch (Chai et al., 2017; Clapham, 2003; Sun and Dong, 2016; Veldhuis et al., 2015; Yu et al., 2016). TRP channels are a major downstream target for GPCR signaling, and the GPCR-TRP axis is vital for pain, neurogenic inflammation, edema and itch (Basbaum et al., 2009; Bautista et al., 2006; Grace et al., 2014; Veldhuis et al., 2015). Second messenger pathways generated from activated GPCRs can alter TRP channel activity and increase the expression of TRP channels at the cell surface. Additionally, GPCR activation can directly activate TRP channels (GPCR-TRP channel coupling) or enhance TRP responsiveness to their activators, a process known as TRP sensitization (Veldhuis et al., 2015).

Mas-related G-protein-coupled-receptors (MrGPRs) are a family of GPCRs that play important roles in various somatosensory functions, are key receptors in itch sensation and also have a role in nociception. MrGPRs are expressed in peripheral sensory neurons, dorsal root ganglion (DRG), and trigeminal ganglion (TG) (Dong et al., 2001; Flegel et al., 2015; Zylka et al., 2003), and have been associated with histamine dependent and histamine-independent itch signaling pathways (Li et al., 2022; Liu et al., 2009; Tseng et al., 2019). Within the MrGPR family, the primate-specific subfamily X, comprised of 4 receptors, has emerged as promising pharmacological targets due to their roles in pain, inflammation, neuroimmune diseases, allergies and itch (Han et al., 2013; Liu et al., 2009; Veldhuis et al., 2015). MrGPRX1, in humans, has been identified as a receptor for pruritogens such as the antimalarial drug chloroquine (CQ). Furthermore, Bovine Adrenal Medulla (BAM) 8–22 peptide, an endogenous pruritogen and proenkephalin A gene product, exhibits the highest specificity for human MrGPRX1 (Guo et al., 2023; Lembo et al., 2002; Li et al., 2017; Liu et al., 2023; Sanjel et al., 2019; Sikand et al., 2011). BAM8-22 binds and activates MrGPRX1 found on primary sensory neurons to transmit itch signals from the periphery to the central nervous system. MrGPRX1 is a Gα_*q*_ coupled GPCR and once activated at the cell surface by extracellular agonists, facilitates intracellular Ca^2+^ release and ion channel activation (Gan et al., 2023; McMillan et al., 2021). Following activation, most GPCRs are endocytosed to the endosomal network. Previous studies have demonstrated that GPCRs can exhibit sustained compartmentalized signaling from intracellular organelles, and this prolonged signaling plays a key role in neuronal activation associated with conditions like chronic pain (Jensen et al., 2017; Jimenez-Vargas et al., 2018). Given the established role of MrGPRX1 in mediating itch, we hypothesize that MrGPRX1 can signal from intracellular compartments, and that endosomal signaling of MrGPRX1 is crucial for sensitizing TRPV1 channels, leading to enhanced itch sensation.

## Results

### BAM8-22 stimulates MrGPRX1 endocytosis and trafficking to endosomes

**FIGURE 1:**
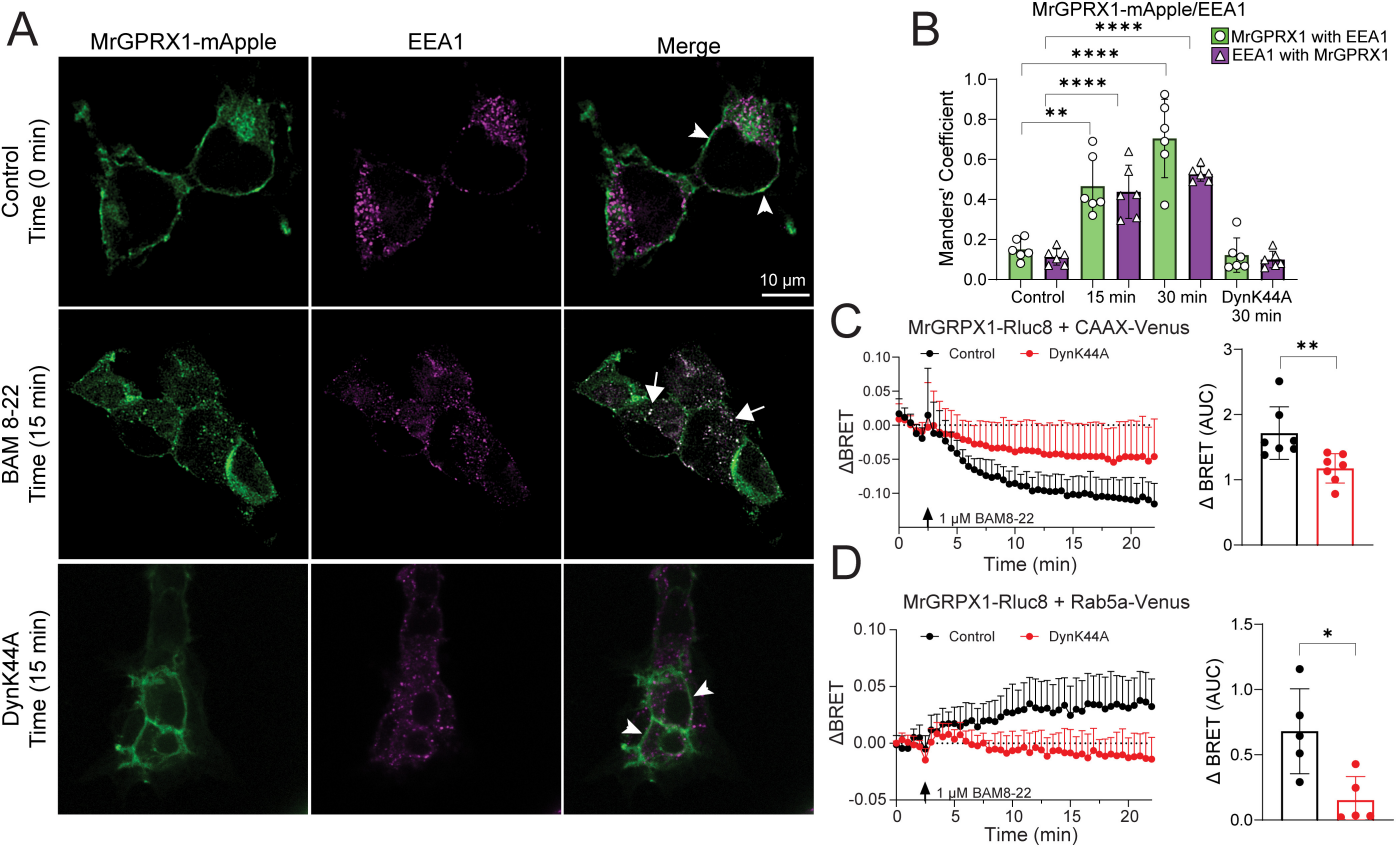
MrGPRX1 endocytosis can be blocked by dynamin inhibition. **(A)** Plasma membrane bound MrGPRX1-mApple is endocytosed to early endosomes (EEA1 label) 15 min following the addition of BAM8-22 in HEK293 cells. MrGPRX1 endocytosis is blocked by the expression of dominant negative mutant of dynamin (DynK44A). Arrowheads, cell-surface; arrows, endosomal MrGPRX1-mApple. Scale 10 μm. Representative images, *n* = 6 independent experiments. **(B)** Manders’ colocalization coefficients show significant increase in the colocalization of MrGPRX1-mApple with EEA1, and EEA1 with MrGPRX1-mApple following stimulation with BAM8-22 that is blocked by co-expression of DynK44A. Each point represents an independent experiment (*n* = 6), *P ≤ 0.05, **P ≤ 0.01, ****P ≤ 0.01. One-Way ANOVA, Dunnet’s multiple comparisons. BAM8-22-induced (1 μM) trafficking of MrGPRX1-Rluc8 from the **(C)** plasma membrane (CAAX-Venus) to **(D)** early endosomes (Rab5a-Venus) in HEK293 cells is blocked by DynK44A expression. Area under the curve (AUC) represents quantification of change in BRET over time. *n* ≥ 5, *P ≤ 0.05, **P ≤ 0.01. Unpaired *t*-test. All data are mean ± SD.

MrGPRX1 trafficking following activation was monitored by specific labeling with mApple, a red fluorescent protein genetically fused to the intracellular C-terminus of the receptor. We transiently expressed MrGPRX1-mApple in HEK293 cells and tracked its internalization using confocal microscopy. In vehicle-treated cells, fluorescent MrGPRX1-mApple was principally localized at the plasma membrane (Figure 1A) (Control; arrowheads). After BAM8-22 treatment (1 μM, 15 min), MrGPRX1-mApple was depleted from the plasma membrane and accumulated in intracellular vesicles (Figure 1A) (BAM8-22; arrows). Vesicles were co-stained with an antibody to the early endosome antigen 1 (EEA1), an established marker for early endosomes (Jensen et al., 2017; Latorre et al., 2022). Thus, BAM8-22 stimulation induced MrGPRX1-mApple trafficking from the plasma membrane to early endosomes. Inhibition of dynamin-mediated endocytosis was accomplished by the overexpression of the dominant negative mutant of dynamin (DynK44A) (Sundborger et al., 2014). Co-expression of DynK44A inhibited agonist-stimulated MrGPRX1-mApple internalization (Figure 1A) (DynK44A; arrowheads). The colocalization of MrGPRX1-mApple with EEA1 was quantified with ImageJ using the JACoP plugin and with Otsu’s thresholding to define signal, yielding Manders’ coefficients across multiple cells. Analysis showed that as early as 15 min post BAM8-22 treatment, ∼45% of early endosomes contained MrGPRX1-mApple. By 30 min, ∼70% of the total receptor population was localized within early endosomes. Receptor trafficking was significantly inhibited by co-expression of DynK44A, with only ∼11% of the early endosomes showing MrGPRX1-mApple presence (Figure 1B).

Bioluminescence resonance energy transfer (BRET) assays were used to quantify the trafficking and colocalization of MrGPRX1 in the endosomal compartment. HEK293 cells were transfected to express MrGPRX1 tagged with the Renilla Luciferase (MrGPRX1-RLuc8) and various subcellular markers to identify the plasma membrane (CAAX-Venus), or early endosomes (Rab5a-Venus) coupled to the BRET acceptor Venus (Jensen et al., 2017). MrGPRX1-RLuc8 expressing cells were stimulated with BAM8-22 (1 μM) and the changes in BRET signal were measured. BAM8-22 activation of MrGPRX1-RLuc8 decreased the BRET signal between MrGPRX1-RLuc8 and the plasma membrane marker (Figure 1C) (CAAX-Venus), and increased the BRET signal between MrGPRX1-RLuc8 and the early endosome marker (Figure 1D) (Rab5a-Venus), denoting receptor internalization and trafficking away from the plasma membrane to early endosomes. Additionally, BAM8-22 stimulation resulted in an increase in BRET signal between MrGPRX1-RLuc8 and the late endosome marker (Supplementary Figures 1A,B) (Rab7a-Venus), and recycling endosomes (Supplementary Figures 1C,D) (Rab11a-Venus). Furthermore, co-expression of DynK44A significantly decreased the agonist-stimulated change in BRET (Figures 1C,D). These results demonstrate that MrGPRX1 traffics from the plasma membrane to the endosomal pathway after BAM8-22 stimulation in a dynamin-dependent process.

### MrGPRX1 endocytosis mediates signaling in subcellular compartments

**FIGURE 2:**
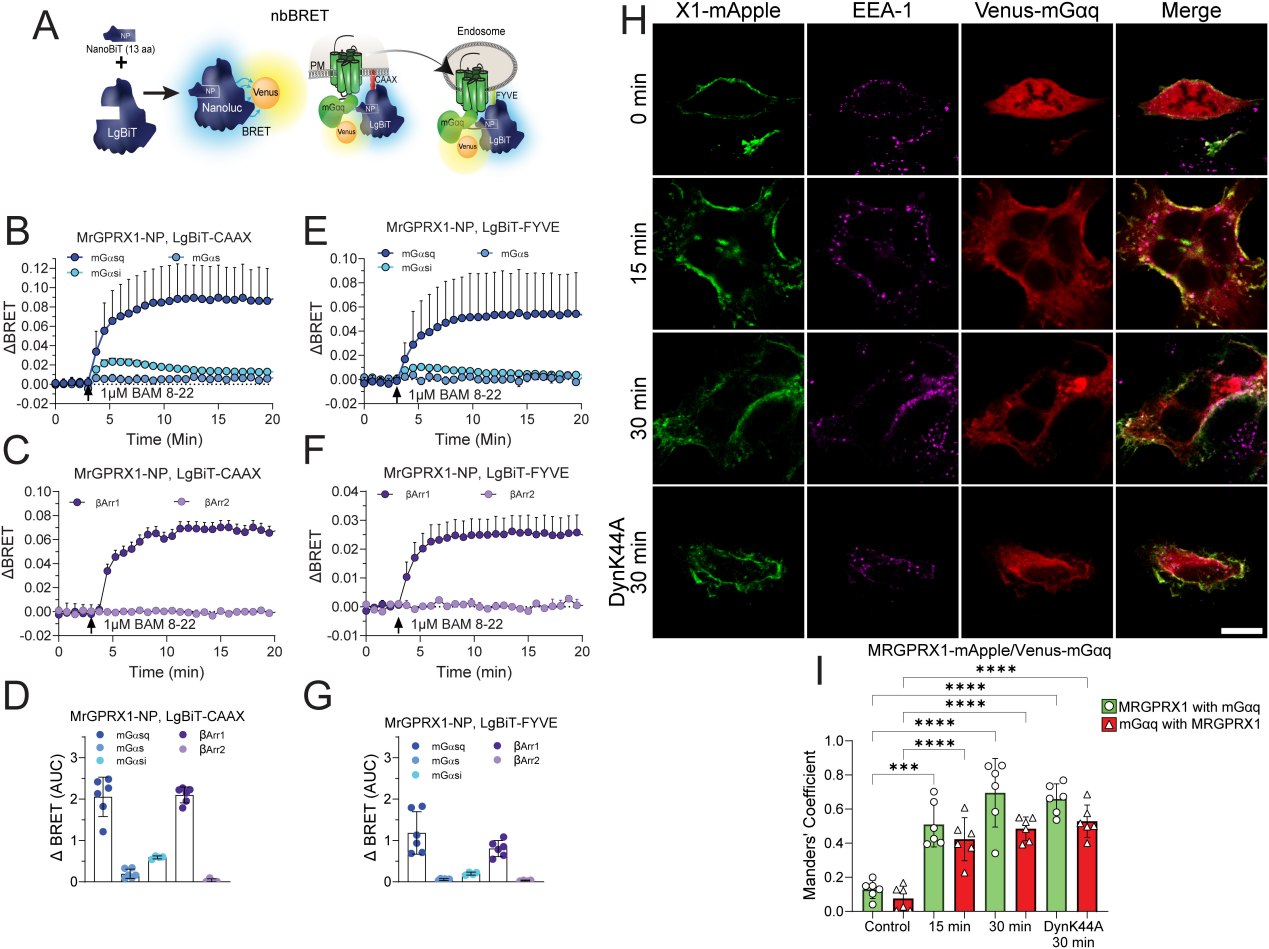
MrGPRX1 recruits miniGα_q_ (mGα_q_) and β-arrestin 1 (βArr1) to plasma membrane and early endosomes. **(A)** Illustration of NanoBit-BRET (nbBRET) assays. nbBRET uses a split luciferase (NanoBit or NP and LgBiT) that when in close proximity form a functional nanoluciferase to detect BRET between three different proteins; receptor (MrGPRX1), effector (mGα_s/q_ or βarr1), and proteins resident in subcellular compartments. Plasma membrane (PM). BAM8-22 induced recruitment of Venus-mGα_q_ to **(B)** the plasma membrane (CAAX-LgBit) and **(E)** early endosomes (FYVE-LgBiT) in HEK293 cells. BAM8-22 induced recruitment of Venus-mGα_i_ to **(B)** the plasma membrane (CAAX-LgBit). BAM8-22 induced recruitment of Venus-βArr1 to **(C)** the plasma membrane (CAAX-LgBit) and **(F)** early endosomes (FYVE-LgBiT) in HEK293 cells. **(D,G)** Area under the curve (AUC) represents quantification of change in nbBRET over time. *n* ≥ 4. **(H)** HEK293 cells express Venus-mGα_q_ at the cytosol and MrGPRX1-mApple on the plasma membrane in quiescent cells. BAM8-22 activation of MrGPRX1-mApple recruits Venus-mGα_q_ and stimulates receptor internalization. Expression of DynK44A inhibited MrGPRX1 endocytosis. Scale 10 μm. Representative images, *n* = 6 independent experiments. **(I)** Manders’ colocalization coefficients show significant increase in the colocalization of MrGPRX1-mApple with Venus-mGα_q_, and Venus-mGα_q_ with MrGPRX1-mApple following stimulation with BAM8-22. Each point represents an independent experiment (*n* = 6), ***P ≤ 0.001, ****P ≤ 0.01. One-Way ANOVA, Dunnet’s multiple comparisons. All data are mean ± SD.

To assess if MrGPRX1 can persist in an active state and recruit β-arrestin isoforms and G proteins to early endosomes, we used Nanobit-BRET (nbBRET) assays. The nbBRET assays were designed to enable the measurement of the interaction between three proteins by splitting the NanoLuc luciferase (NLuc) into two peptides, a small 13 amino acid peptide known as NanoBiT fragment (NP) and a larger fragment called LgBiT (Dixon et al., 2016; Hegron et al., 2023). When these two peptides are in close proximity ( < 10 nm), they form a functional NLuc luciferase that can emit luminescence in the presence of its substrate furimazine. nbBRET has been used to characterize the recruitment of G proteins by GPCRs (i.e., Neurokinin 1 Receptor, Calcitonin Like Receptor and Protease Activated Receptor 2) to various subcellular compartments (Hegron et al., 2023; Peach et al., 2026; Teng et al., 2025). This approach allows us to measure the ability of MrGPRX1 to recruit effector proteins to different subcellular compartments. For this, we transfected HEK293 cells with a C-terminal tagged MrGPRX1 with NP (MrGPRX1-NP) and either a plasma membrane marker (CAAX-LgBiT) or an early endosome marker (FYVE-LgBiT), and different effector proteins coupled to the BRET acceptor Venus (Figure 2A).

We first evaluated the recruitment of G proteins to MrGPRX1-NP. For this, we used miniGα proteins (mGα) which are N-terminally truncated Gα isoforms that can bind to active conformations of GPCRs (Nehmé et al., 2017; Wan et al., 2018). It has been reported that MrGPRX1 primarily recruits and associates with Gα_*q*_ (Gan et al., 2023; Guo et al., 2023; Tseng et al., 2019). Additionally, a few studies have suggested Gα_*i*_ recruitment following BAM8-22-mediated activation of the receptor (Guo et al., 2023; Li et al., 2017). Consistent with this, stimulation with BAM8-22 (1 μM) caused an increase in nbBRET signal at the plasma membrane between MrGPRX1-NP, CAAX-LgBiT and Venus-mGα_*q*_, and Venus-mGα_*i*,_ but not Venus-mGα_*s*_ (Figures 2B,D). When the endosomal marker FYVE-LgBiT was transfected, an increase in nbBRET in response to BAM8-22 (1 μM) was detected between MrGPRX1-NP and Venus-mGα_*q*_, but not Venus-mGα_*i*_ or Venus-mGα_*s*_ (Figures 2E,G), showing that Gα_*q*_ proteins can be recruited to endosomal compartments containing activated MrGPRX1. Additionally, BAM8-22 (1 μM) activation induced the recruitment and association of β-arrestin 1-Venus, but not β-arrestin 2-Venus, with MrGPRX1-NP at the plasma membrane (Figures 2C,D). A similar pattern was observed at early endosomes, where BAM8-22 (1 μM) induced an increase in nbBRET between MrGPRX1-NP, FYVE-LgBiT and β-arrestin 1-Venus but not β-arrestin 2-Venus (Figures 2F,G). Taken together, these results show that following activation by BAM8-22, MrGPRX1 is bound by β-arrestin 1, undergoes dynamin-dependent endocytosis and traffics to early endosomes where activated MrGPRX1 can recruit effector proteins like mGα_*q*_.

Confocal microscopy was used to confirm the translocation of mGα_*q*_ to the plasma membrane and early endosomes in response to BAM8-22-mediated activation of MrGPRX1. In unstimulated HEK293 cells, Venus-mGα_*q*_ was primarily detected in the cytosol (Figure 2H) (0 min). After 15 min of BAM8-22 stimulation, MrGPRX1-mApple was observed in EEA1-positive early endosomes (Figure 1A), and Venus-mGα_*q*_ had translocated to the plasma membrane and early endosomes (Figure 2H) (15 min). Manders’ coefficient analysis revealed that ∼50% of MrGPRX1-mApple co-localized with Venus-mGα_*q*_ (Figure 2I) (15 min). By 30 min, MrGPRX1-mApple remained localized in early endosomes (Figure 1B), where ∼69% of the total receptor population co-localized with Venus-mGα_*q*_ (Figures 2H,I) (30 min). When endocytosis was inhibited via DynK44A co-expression, MrGPRX1-mApple was sequestered at the plasma membrane, where ∼65% of the receptor co-localized with Venus-mGα_*q*_ (Figures 2H,I) (DynK44A 30 min). Together, these results demonstrate that MrGPRX1 recruits mGα_*q*_ to both the plasma membrane and early endosomes.

**FIGURE 3:**
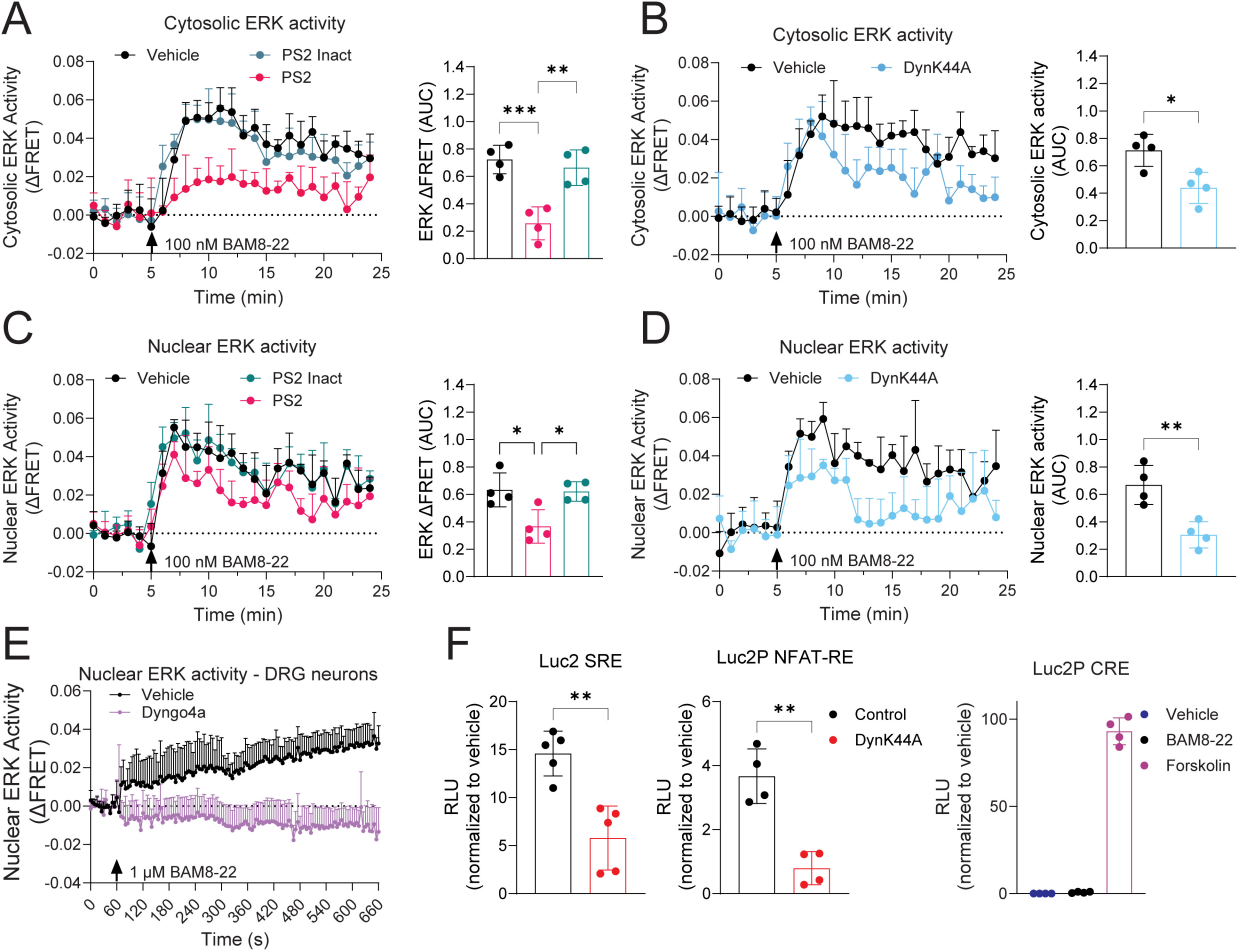
Characterization of the compartmentalized subcellular signaling of MrGPRX1. ERK phosphorylation was determined using EKAR FRET biosensors. BAM8-22 induced activation of cytosolic **(A,B)** and nuclear **(C,D)** ERK in HEK293 cells. Pretreatment with endocytic inhibitor PS2 **(A,C)** or expression of DynK44A **(B,D)** attenuate ERK activation. Area under the curve (AUC) represents quantification of ERK activation over time. *n* = 4, *P ≤ 0.05, **P ≤ 0.01, ***P ≤ 0.001. One-Way ANOVA, Tukey’s multiple comparisons. Unpaired *t*-test. **(E)** Pretreatment with the dynamin inhibitor Dyngo4a reduced nuclear ERK activation induced by BAM8-22 in mouse DRG neurons. **(F)** Luciferase transcriptional assays of HEK293 cells expressing MrGPRX1 and Luc2-SRE, Luc2-NFAT-RE, or Luc2-CRE in response to BAM8-22 (1 μM, 4 h). Expression of DynK44A attenuated MrGPRX1-induced transcription. Relative luminescence units (RLU). *n* ≥ 4, **P ≤ 0.01. Unpaired *t*-test. All data are mean ± SD.

Given the important role of ERK signaling in regulating crucial cellular responses, coupled with previous studies that demonstrated endosomal GPCR signaling differentially regulated ERK activity (Hegron et al., 2023; Jensen et al., 2017; Teng et al., 2025), we investigated the contribution of MrGPRX1 endocytosis to ERK activation. This was monitored with high spatial and temporal resolution using Förster resonance energy transfer (FRET) based ERK biosensors known as EKAR biosensors. These biosensors contain a reversible substrate sequence flanked by two fluorophores and can be targeted to different subcellular compartments (Halls et al., 2015; Harvey et al., 2008). We measured cytosolic (CytoEKAR) and nuclear (NucEKAR) ERK activity in HEK293 cells expressing human MrGPRX1. BAM8-22 induced robust and sustained ERK activation in the cytosol (Figures 3A,B) and nucleus (Figures 3C,D). Interestingly, pretreatment with the clathrin inhibitor Pitstop2 (PS2) significantly decreased BAM8-22-induced activation of both cytosolic (Figure 3A) and nuclear (Figure 3C) ERK. This inhibition was not observed when the inactive analog of Pitstop2 (PS2 Inact), a structural derivative of PS2 that lacks the ability to inhibit clathrin-mediated endocytosis, was used (Figures 3A,C). Furthermore, in cells expressing DynK44A, ERK activity was significantly reduced in both compartments compared to the control condition (Figures 3B,D). Given that MrGPRX1 is mainly expressed on primary sensory neurons, we used an adeno-associated virus (AAV-NucEKAR) to express the ERK biosensor specifically in the nucleus of mouse dorsal root ganglion (DRG) neurons (Supplementary Figure 2). Similar to our results in HEK293 cells, treatment with BAM8-22 (1 μM) produced a sustained nuclear ERK response in mouse DRG neurons. This activity was prevented by preincubation with the dynamin inhibitor, Dyngo4a (10 μM) (Figure 3E). Thus, these results highlight the necessity of endosomal trafficking and signaling of MrGPRX1 in driving a complete cellular response to MrGPRX1 activation.

Nuclear ERK activation is a crucial step in the regulation of immediate early response genes that guide alterations in neuronal plasticity and neuronal networks (Fowler et al., 2011; Wiegert and Bading, 2011). We hypothesized that the activation of nuclear ERK via the endosomal signaling of MrGPRX1 would alter gene expression patterns that alter cellular responses. To further investigate the role of endosomal MrGPRX1 on gene expression, we used transcriptional luciferase reporter assays (Cheng et al., 2010). HEK293 cells were co-transfected with MrGPRX1 and luciferase reporter vectors. These vectors contain the luciferase gene (Luc2P) driven by promoters featuring specific response elements (REs). BAM8-22 (1 μM) induced transcriptional activity for elements responsive to both the ERK signaling pathway (Figure 3F) (Luc2P-serum response element, SRE), and Gα_*q*_ activation (Figure 3F) (Luc2P Nuclear Factor of Activated T-cells response element, NFAT-RE). Moreover, the co-expression of DynK44A significantly decreased these transcriptional activities (Figure 3F). Consistent with previous reports, BAM8-22 failed to induce activity in response to cAMP (Figure 3F) (Luc2P cAMP response element, CRE) compared to Forskolin (10 μM) used as a positive control (Figure 3F). These findings, together with the evidence of nuclear ERK signaling, establish MrGPRX1 endocytosis and subsequent endosomal signaling as key requirements for generating the full cellular response to BAM8-22.

### MrGPRX1 sensitization of TRPV1 channels is regulated by PKC

**FIGURE 4:**
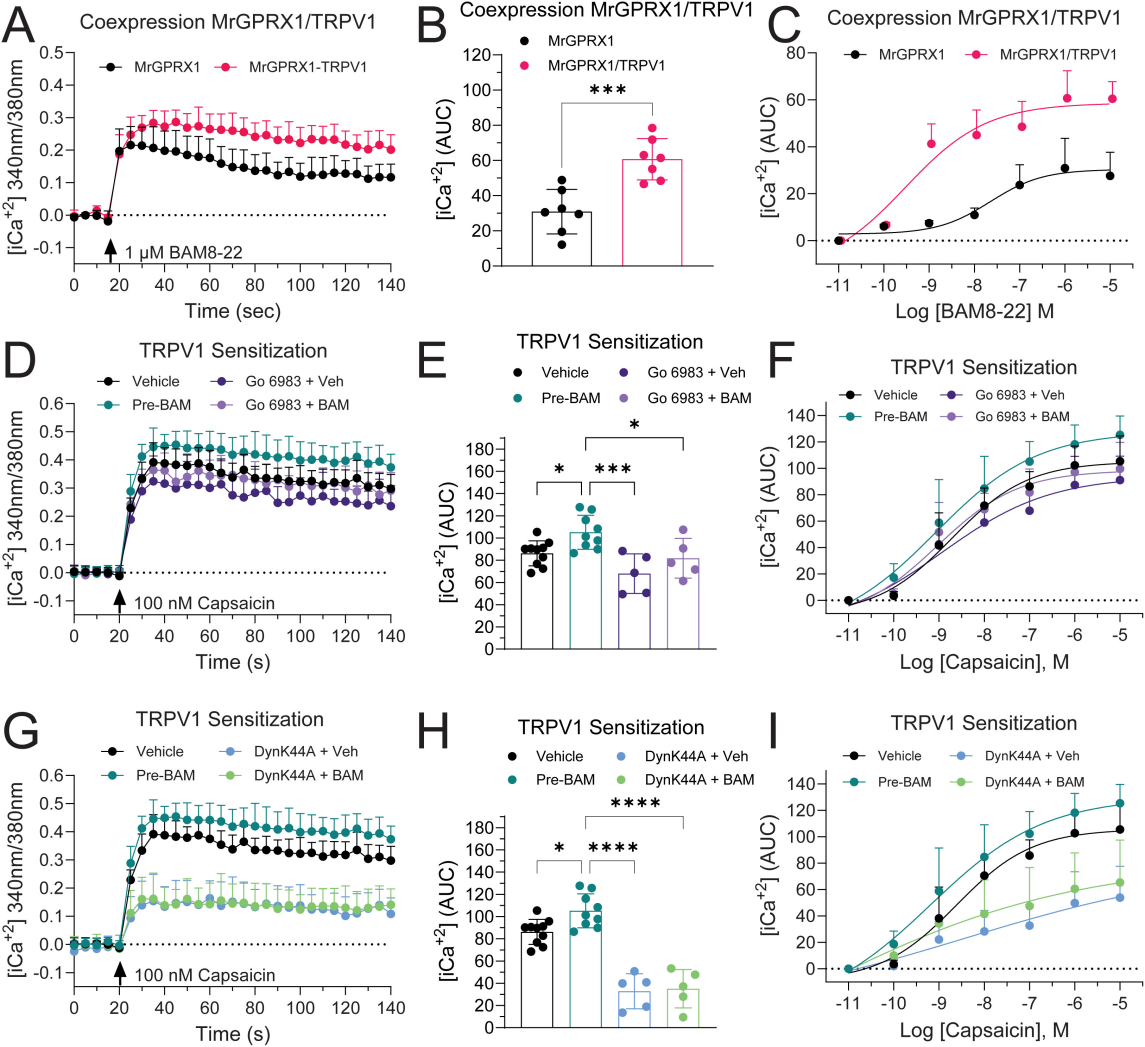
MrGPRX1 modulates TRPV1 channels. **(A)** BAM8-22-evoked Ca^2+^ signaling in HEK293 cells expressing MrGPRX1 alone or with TRPV1 channels. Area under the curve (AUC) represents quantification of intracellular Ca^2+^ change over time **(B)** and in a dose dependent manner **(C)**. *n* = 7, ***P ≤ 0.001. Unpaired *t*-test. **(D)** Capsaicin-evoked Ca^2+^ influx in HEK293 cells expressing MrGPRX1 and TRPV1. MrGPRX1-mediated TRPV1 sensitization is decreased by pre-treatment with PKC inhibitor Gö 6983. Area under the curve (AUC) represents quantification of intracellular Ca^2+^ influx over time **(E)** and in a dose dependent manner **(F)**. *n* ≥ 5, *P ≤ 0.05, ***P ≤ 0.001. One-Way ANOVA, Tukey multiple comparisons. **(G)** Expression of DynK44A blocked MrGPRX1-mediated TRPV1 sensitization. Quantification of intracellular Ca^2+^ influx over time (AUC) **(H)**, and in a dose dependent manner **(I)**. *n* ≥ 5, *P ≤ 0.05, ****P ≤ 0.01. One-Way ANOVA, Tukey multiple comparisons. All data are mean ± SD.

TRPV1 channels play crucial roles in itch transmission (Sun and Dong, 2016). Previous studies have shown that MrGPRX1 can sensitize TRPV1 channels via Gα_*q*_-induced activation of protein kinase C (PKC) (Solinski et al., 2012). To corroborate this, we measured changes in intracellular Ca^2+^ levels [(iCa^2+^)c] using calcium imaging. BAM8-22 produced an increase in (iCa^2+^) in HEK293 cells expressing MrGPRX1. This calcium response was significantly enhanced by the co-expression of TRPV1 channels (Figures 4A–C), confirming functional coupling between MrGPRX1 and TRPV1. Furthermore, as expected, pre-stimulation with BAM8-22 prior to capsaicin application significantly potentiated TRPV1-mediated Ca^2+^ influx. This effect was prevented by pre-treatment with the PKC inhibitor Gö 6983 (100 nM) (Figures 4D–F). Notably, this MrGPRX1-mediated TRPV1 sensitization was also abolished when endocytosis was blocked by the co-expression of DynK44A (Figures 4G–I). Thus, BAM8-22-mediated activation of MrGPRX1 sensitizes TRPV1 channels in a PKC-dependent manner and is driven by endosomal signaling.

### MrGPRX1 sensitization of TRPV1 channels in DRG neurons

**FIGURE 5:**
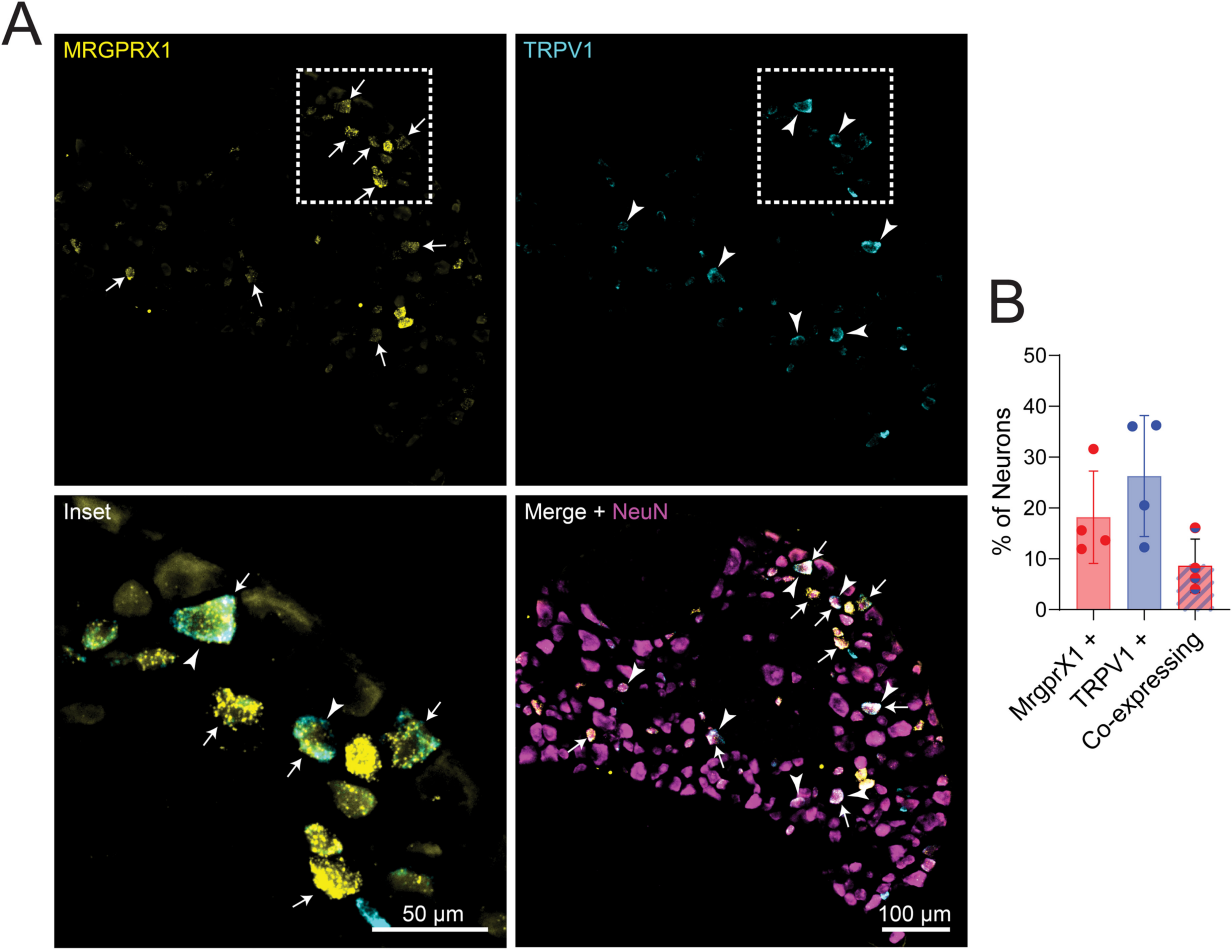
Characterization of MrGPRX1 and TRPV1 expression in mouse dorsal root ganglion (DRG) neurons. **(A)** Representative RNAScope^®^ image of the detection of MrGPRX1 and TRPV1 mRNA in mouse DRG neurons identified by NeuN immunofluorescence. Arrows represent MrGPRX1 positive neurons, and arrowheads represent TRPV1 positive neurons. Scale, 100 μm, and 50 μm in inset. **(B)** Quantified percentages of MrGPRX1 positive neurons, TRPV1 positive neurons, and co-expression of MrgprX1 and TRPV1 in DRG neurons. *N* = 4 mice. All data are mean ± SD.

To determine if MrGPRX1 also sensitizes TRPV1 channels in primary sensory neurons, we first confirmed their expression and co-localization in mouse DRGs. Although the individual expression of MrGPRX1 and TRPV1 in these neurons is well documented, their precise co-localization has not been previously reported. For this, we used RNAscope^®^ in situ hybridization. *Mrgprx1* (MrGPRX1) and *Trpv1* (TRPV1) mRNAs were detected in the same DRG neurons, identified by NeuN immunostaining (Figure 5A). *Mrgprx1* was detected in ∼18% and *Trpv1* in ∼26% of neurons (Figure 5B). *Mrgprx1* and *Trpv1* were co-expressed in ∼8% of total mouse DRG neurons (Figure 5B).

**FIGURE 6:**
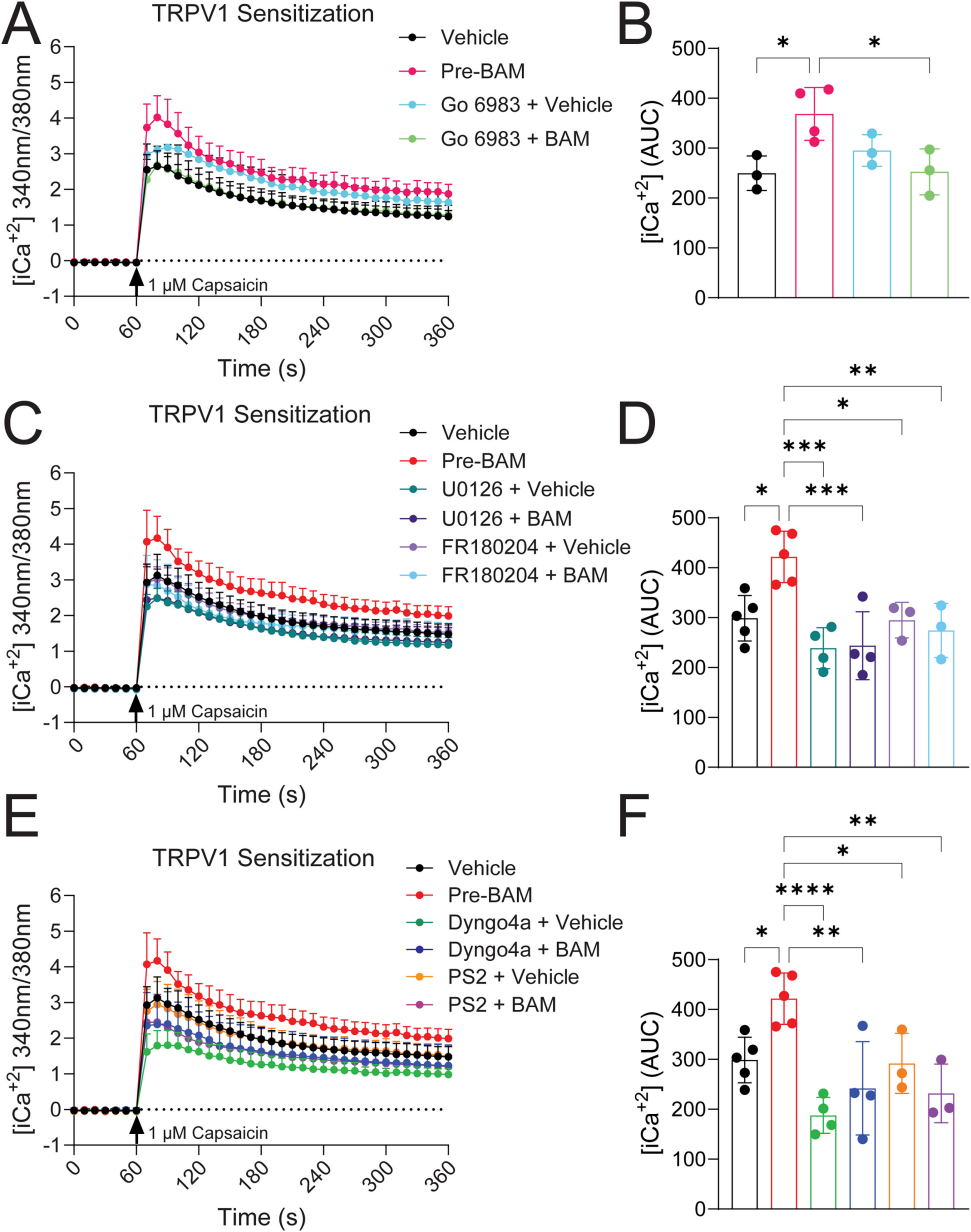
MrGPRX1 sensitizes TRPV1 channels in mouse DRG neurons. Capsaicin-evoked Ca^2+^ influx in mouse DRG neurons. BAM8-22-mediated TRPV1 sensitization is decreased by pre-treatment with PKC inhibitor Gö 6983 **(A,B)**, MEK inhibitor U0126 (C-D), ERK inhibitor FR 180204 **(C,D)**, and endocytic inhibitors Dyngo4a or PS2 **(E,F)**. Area under the curve (AUC) represents quantification of intracellular Ca^2+^ influx over time. *n* ≥ 3 mice, *P ≤ 0.05, **P ≤ 0.01, ***P ≤ 0.001, ****P ≤ 0.01. One-Way ANOVA, Tukey multiple comparisons. All data are mean ± SD.

To further validate MrGPRX1 sensitization of TRPV1 channels, we performed live cell Ca^2+^ imaging using isolated mouse DRG neurons. Results showed that pre-treatment with BAM8-22 (1 μM) significantly increased capsaicin-induced calcium influx through TRPV1 (Figures 6A–F). Similar to what we observed in HEK293 cells, this increased Ca^2+^ influx was prevented by pre-incubation with the PKC inhibitor Gö 6983 (100 nM) (Figures 6A,B). These results confirm that PKC activation is essential for MrGPRX1 mediated sensitization of TRPV1 channels in DRG neurons.

In addition, we investigated the role of ERK signaling in TRPV1 sensitization in response to BAM8-22. For this, we pre-incubated the neurons with the selective mitogen-activated extracellular signal-regulated kinase (MEK) inhibitor U0126 (10 μM; 30 min) to inhibit the MEK/ERK signaling cascade. Calcium imaging results demonstrated that TRPV1 sensitization by BAM8-22 was significantly blocked by the pre-incubation with U0126 (Figures 6C,D). Furthermore, to confirm that ERK activity regulates TRPV1 sensitization, we also tested the selective ERK inhibitor FR 180204 (30 μM; overnight). The results showed that ERK inhibition partially prevented the sensitization of TRPV1 channels (Figures 6C,D). These findings indicate that ERK activation, in conjunction with PKC activation, regulate BAM8-22-mediated sensitization of TRPV1 channels.

Next, we investigated the role of endosomal MrGPRX1 signaling in TRPV1 sensitization. For this, we pre-incubated mouse cultured neurons with the endocytic inhibitors Dyngo4a (10 μM) or PS2 (10 μM) and then performed calcium imaging. Both inhibitors effectively suppressed the BAM8-22 induced sensitization of TRPV1 channels (Figures 6E,F), validating our hypothesis that endosomal signaling of MrGPRX1 is crucial for this process in DRG neurons.

### Endosomal MrGPRX1 signaling mediates scratching behaviors

**FIGURE 7:**
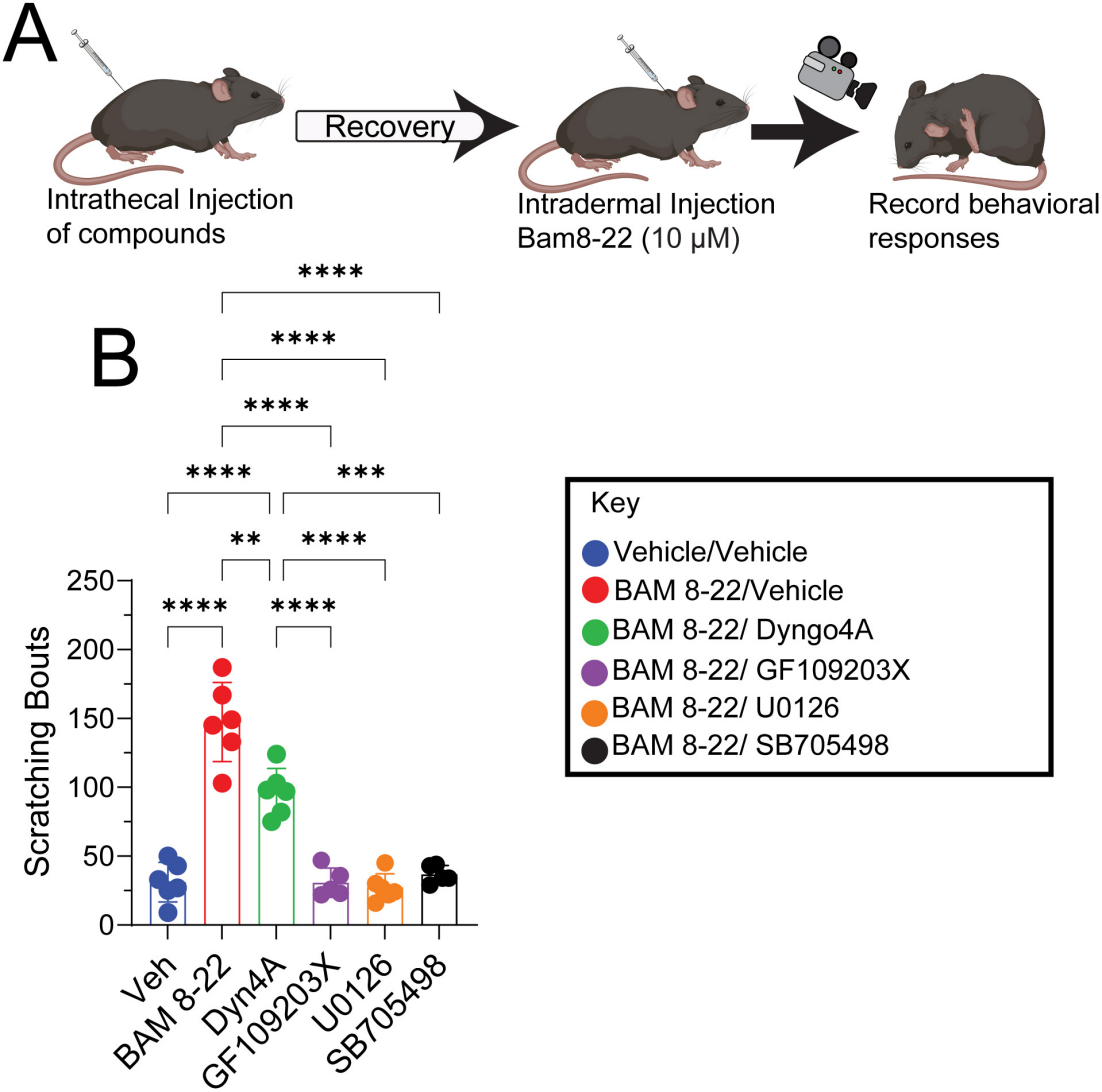
Effect of endocytic, MEK, PKC and TRPV1 inhibitors in BAM8-22 induced scratching responses in mice. **(A)** Illustration of the experimental protocol. **(B)** Scratching bouts induced by vehicle or BAM8-22 intradermal injection into the nape of the mouse 30 min after intrathecal administration of dynamin inhibitor Dyngo4A, MEK inhibitor U0126, PKC inhibitor GF109203X, or TRPV1 inhibitor SB705498. *n* ≥ 5 mice, **P ≤ 0.01, ***P ≤ 0.001, ****P ≤ 0.0001. One-Way ANOVA, Tukey multiple comparisons. All data are mean ± SD.

Calcium imaging results confirmed that BAM8-22 sensitization of TRPV1 is regulated by MrGPRX1 endosomal signaling and both MrGPRX1 and TRPV1 have been shown to control scratching behavior in mice (Li et al., 2022; Yu et al., 2016). To evaluate the role of endosomal MrGPRX1 signaling in scratching behavior, we first injected the endocytic inhibitor Dyngo4a or vehicle control intrathecally. Mice received a single intrathecal injection and were allowed to recover for 30 min before testing. We then examined the scratching behavior in response to an intradermal injection of BAM8-22 (10 μM) into the nape of the mouse. Scratching behavior, grooming, distance traveled, and inactivity were monitored using the Behavioral Spectrometer (Figure 7A and Supplementary Figure 3). BAM8-22 administration caused a significant increase in total scratching bouts (Figure 7B) compared to vehicle-injected mice. Interestingly, pre-administration of Dyngo4a (30 μM) resulted in a significant decrease in scratching bouts in response to BAM8-22 compared to vehicle-treated mice (Figure 7B). To evaluate whether this scratching behavior was partially mediated by PKC activity and MEK/ERK signaling, we intrathecally injected the PKC inhibitor GF109203X (0.25 μg/5 μL) or MEK inhibitor U0126 (0.5 μg/5 μL) 30 min before assessing the scratching response to intradermal injection of BAM8-22 at the nape. Notably, pre-administration of these inhibitors suppressed the scratching response to BAM8-22 (Figure 7B). Finally, the TRPV1 antagonist SB705498 (0.05 μg/5 μL) was intrathecally injected to confirm the role of these channels in the BAM8-22-induced scratching response. As expected, antagonizing TRPV1 channels caused a decrease in the scratching response to BAM8-22 when compared to the vehicle-treated group (Figure 7B).

Grooming, total distance traveled, and inactivity were not significantly altered by BAM8-22 administration. Furthermore, there was no significant change in grooming, distance traveled, and inactivity in the Dyngo4a treated group (Supplementary Figure 3). However, grooming behavior was significantly decreased in groups injected with the PKC inhibitor (GF109203X) or the TRPV1 antagonist (SB705498) (Supplementary Figure 3A). Additionally, time spent inactive was significantly increased in mice treated with PKC inhibitor (GF109203X), MEK inhibitor (U0126), or TRPV1 antagonist (SB705498) when compared with vehicle-treated mice (Supplementary Figure 3C).

Collectively, these results are consistent with our hypothesis that internalization and endosomal signaling of MrGPRX1 in response to BAM8-22 are important for evoking a scratching response in mice, potentially through PKC- and ERK-mediated sensitization of TRPV1.

## Discussion

This study characterizes a previously unknown signaling mechanism for the human MrGPRX1 receptor. For the first time, our results demonstrate that MrGPRX1, upon stimulation with BAM8-22, binds to β-arrestin-1 and undergoes clathrin- and dynamin-dependent endocytosis. Additionally, these studies show that MrGPRX1 is able to recruit β-arrestin-1 and Gα_*q*_ to the endosomal compartment where endosomal MrGPRX1 regulates both cytosolic and nuclear ERK activation. These data confirm the known PKC-dependent MrGPRX1-mediated TRPV1 sensitization and unveil a new mechanism whereby endosomal signaling of MrGPRX1 regulates TRPV1 sensitization through a MEK/ERK dependent pathway. Supporting these mechanistic *in vitro* results, we found that BAM8-22-induced scratching behaviors in mice are significantly attenuated when either endocytosis of MrGPRX1 or PKC/ERK signaling is impaired. Overall, these results characterize a new signaling mechanism in which BAM8-22-induced activation of MrGPRX1 drives endosomal signaling that regulates TRPV1 sensitization and itch transmission in primary sensory neurons.

The link between prolonged intracellular GPCR signaling events and pathological states, such as chronic pain and itch, is being increasingly emphasized in new studies (Jensen et al., 2017; Jimenez-Vargas et al., 2020; Latorre et al., 2022; Teng et al., 2025). Our findings support this emerging paradigm by demonstrating that the MrGPRX1 receptor, implicated in pruritus, undergoes agonist-induced endocytosis and signals from endosomes. In contrast, a previous report suggested that the human MrGPRX1 receptor is resistant to agonist-induced endocytosis (Solinski et al., 2010). This discrepancy is likely attributable to important methodological differences used to study receptor internalization. First, the previous study utilized expression vectors generated from the SNSR4 gene, which represents an earlier sequence version of MrGPRX1. Second, in that report, MrGPRX1 cell surface levels were detected using ELISA experiments where cells were immediately transferred to ice following stimulation with BAM8-22. Given that low temperatures are known to inhibit endocytic pathways (Szewczyk-Roszczenko et al., 2023), this reported lack of receptor endocytosis may be a methodological artifact. By comparison, our study employed several rigorous qualitative and quantitative techniques, including BRET and confocal microscopy, to confirm that MrGPRX1 undergoes endocytosis following activation. Furthermore, the observations that BAM8-22-mediated activation of MrGPRX1 results in Gα_*q*_ and β-arrestin-1 recruitment to early endosomes and that MrGPRX1-induced transcription was decreased upon blocking endocytosis provide functional evidence that MrGPRX1 continues to signal from early endosomes, thereby directly linking MrGPRX1 to the formation of signalosomes and providing a mechanism for prolonged intracellular signaling.

Sensitization of peripheral sensory neurons is a key factor in the pathophysiology of chronic pruritus. This peripheral sensitization can lead to alloknesis, defined as the abnormal sensation where an innocuous stimulus triggers an itch sensation, and hyperknesis, defined as an excessive itch sensation to pruritic stimuli, which are common symptoms in patients with chronic pruritus conditions (Andersen et al., 2018; Lamotte, 1988; LaMotte, 1992). Unlike transient pruritus, chronic pruritus relies on sustained signaling and sensitization of sensory neurons. Our data demonstrates that MrGPRX1 endosomal signaling is one mechanism that leads to this sustained activity, including prolonged nuclear ERK activation, which is a known driver of neuronal plasticity and long-term sensitization (Cheng and Ji, 2008; Ji et al., 2002; Wiegert and Bading, 2011). This finding suggests that the spatial location of MrGPRX1 signaling may determine the nature of the itch response. This mechanism offers a therapeutic opportunity through biased agonism: by selectively targeting the endosomal signaling of MrGPRX1 while leaving plasma membrane signaling intact, we could potentially treat chronic itch while avoiding unnecessary side effects.

These results are consistent with previous reports that implicate transient receptor potential (TRP) channels, particularly TRPA1, as key downstream effectors in MrGPR-mediated itch signaling. Research confirms that TRPA1 is required for itch responses triggered by MrGPR agonists, including chloroquine and BAM8-22 (Wilson et al., 2011). Furthermore, animal models have established the involvement of TRP family members, including TRPA1 and TRPV1, in both acute and chronic itch. TRPV1 specifically has been shown to be involved in both histaminergic and non-histaminergic itch transmission (Fiebig et al., 2023; Shim et al., 2007). Notably, TRPV1 was found to be involved in itch signaling induced by BAM8-22 in humans (Fiebig et al., 2023). Other studies have highlighted the involvement of different ion channels in MrGPR-mediated itch. Voltage-gated sodium channels have been shown to have an important role in inducing scratching responses. Particularly, one study found that BAM8-22 induced activation of MrGPRX1 generated inward sodium currents by lowering the activation threshold of TTX-resistant voltage-gated sodium channels. Consistent with this, knockout of Na_*V*_1.9 channels, but not TRPA1, significantly decreased scratching behaviors evoked by BAM8-22 (Tseng et al., 2019).

Our finding that MrGPRX1 signals from endosomes provides the mechanistic basis for its role in regulating TRPV1 activity. The sensitivity to a ligand, and the magnitude and duration of TRP activation, can be augmented by functional interactions with GPCRs, often termed “coupling.” This coupling is generally bidirectional, with the functional interaction of a GPCR and an ionotropic channel like a TRP sometimes leading to an augmentation of GPCR signaling as well (Solinski et al., 2012; Veldhuis et al., 2015). GPCRs typically modulate TRP activity via two general mechanisms. The first involves Gα-mediated activation of phospholipase C (PLC) and phospholipase A2 (PLA2), which cleave fatty acids and generate endogenous TRP activators such as arachidonic acid (AA) and phosphatidylinositol 4,5-bisphosphate (PIP2). In this sense, research indicates that BAM8-22-mediated activation of MrGPRX1 can directly activate TRPV1 channels and this mechanism is driven by PIP2/DAG (Solinski et al., 2012). The second mechanism is through activation of PKC and PKA, serine/threonine kinases that phosphorylate the intracellular C-tail of TRP channels to enhance cell surface expression and activity (Amadesi et al., 2004; Amadesi et al., 2006; Geppetti et al., 2015; Meents et al., 2017; Peng et al., 2020; Poole et al., 2013; Premkumar and Ahern, 2000; Veldhuis et al., 2015; Zhao et al., 2014). TRP channels such as TRPV1 and TRPA1 are widely implicated in transducing GPCR activation into membrane depolarization in nociceptive neurons (Geppetti et al., 2015; Gouin et al., 2017). Consistent with this established framework, our calcium imaging results demonstrate a coupling between MrGPRX1 and TRPV1, showing that MrGPRX1 can modulate TRPV1 activity through activation of PKC. Importantly, RNAScope *in situ* hybridization analysis provided anatomical evidence for this interaction, demonstrating that the mRNA for MrGPRX1 and TRPV1 co-localizes in a subset of DRG neurons. Furthermore, our data show that MrGPRX1 endocytosis and ERK activation are also critical to this sensitization mechanism, pointing to a novel role for prolonged MrGPRX1 endosomal signaling in driving this pathway. However, a critical gap remains: the signaling partners recruited with MrGPRX1 to the endosomal compartment, which result in sustained ERK activation and TRPV1 sensitization, remain to be elucidated. Interestingly, MrGPRX1 was recently shown to form heterodimers with the μ-opioid receptor (OPRM1) in sensory neurons, shifting OPRM1 signaling from Gαi to Gαq to drive PLC-mediated calcium mobilization and enhance opioid-induced itch (Sanjel et al., 2026). This crosstalk could synergistically amplify the PKC-dependent mechanism proposed in our study. However, how MrGPRX1 endosomal signaling specifically functions within this heteromeric complex warrants future investigation. Given that PKC activation typically occurs at the plasma membrane (Igumenova, 2015), we hypothesized that the fast sensitization of TRPV1 channels due to PKC activation provides the initial acute itch response, while the endosomal signaling mediated by ERK activation provides the sustained sensitization that is crucial for the pathophysiology of chronic pruritus.

MrGPRX1 is exclusively expressed in humans and lacks a single-gene mouse ortholog. While it exhibits some sequence homology to murine MrgprA receptors (Dong and Dong, 2018), functional homology is instead shown by other rodent receptors. Specifically, MrgprA3 and MrgprC11 are considered functional orthologs of MrGPRX1 simply because they share the same ligands, chloroquine (CQ) and BAM8-22, respectively (Liu et al., 2009). While our *in vitro* work utilizes heterologous cells expressing human MrGPRX1, our *ex vivo* DRG and *in vivo* behavioral experiments rely on endogenous mouse circuitry. Although this species difference represents a significant limitation for direct translational research, studies using mouse models have nevertheless provided fundamental insights into the *in vivo* function of MrGPRX1 signaling pathway. Notably, recent studies using “humanized” mouse models, in which the endogenous murine MrGPRs gene clusters were replaced by the human MrGPRX1 gene, have provided the foundational insights into how this receptor mediates pain and non-histaminergic itch responses (Li et al., 2017; Tseng et al., 2019). Beyond species differences, other limitations of our study include the use of pharmacological inhibitors of endocytosis and important signaling pathways such as PKC and MEK. These inhibitors could have possible off-target actions, meaning the observed effects might be due to disrupted signaling of other receptors unrelated to MrGPRX1 signaling itself. Supporting this concern, our results showed that administration of PKC or MEK inhibitors altered some non-evoked behaviors, and previous reports have shown a decrease in locomotor activity after PKC inhibition with tamoxifen (Abrial et al., 2014). Whereas the effect of TRPV1 antagonist administration on non-evoked behaviors could be explained by its role in body-temperature maintenance as it has been shown that antagonizing these receptors can cause hyperthermia (Gavva, 2008; Romanovsky et al., 2009). Further studies employing genetic manipulation methods, such as conditional knockouts and the utilization of a “humanized” MrGPRX1 mouse line, will be necessary to validate the specific role of MrGPRX1 and its downstream signaling molecules in these complex *in vivo* behaviors.

In conclusion, we have demonstrated that, after activation, the human MrGPRX1 receptor traffics to endosomes and transmits sustained signals that regulate TRPV1 sensitization underlying itch transmission. Endosomal GPCR signaling presents new challenges for understanding how GPCR signal transduction is regulated and how it contributes to disease states like chronic pruritus, while also opening new targets for therapeutic development.

## Materials and methods

### Drugs and reagents

BAM8-22, GF 109203X (GFX), U0126, FR 180204, Gö 6983, and SB705498 were purchased from Tocris Bioscience (Bristol, UK). DMEM, Hank’s balanced salt solution (HBSS), and ProLong Glass, were purchased from Thermofisher (Waltham, MA). Coelenterazine h was purchased from Nanolight Technology (Pinetop, AZ). Nano-Glo^®^ Luciferase substrate furimazine from Promega (Madison, United States). Fura2AM was purchased from Cayman Chemicals (Ann Arbor, MI). Dyngo 4a was purchased from Abcam (Cambridge, United Kingdom). Dyngo4a inactive was provided by Dr. Adam McCluskey (University of Newcastle, AU). All other reagents were from Sigma (St. Louis, MO) unless otherwise specified.

All the catalog numbers and RRIDs can be found in Supplementary Table 1.

### cDNAs

Constructs for human MrGPRX1 (MrGPRX1-mApple, MrGPRX1-NP) were purchased from Twist Bioscience (San Francisco, CA, United States). The following plasmids and FRET biosensors were purchased from Addgene: CytoEKAR Cerulean/Venus (Cytosolic ERK sensor), NucEKAR Cerulean/Venus (Nuclear ERK sensor), and Dynamin K44A (dominant negative dynamin-1). The pGL4.33(luc2P/SRE/Hygro), pGL4⋅30(luc2P/NFAT-RE/Hygro), and pGL4⋅29 (luc2P/CRE/Hygro) vectors were purchased from Promega (Madison, United States).

For BRET studies, human MrGPRX1 construct was modified with Renilla^®^ luciferase 8 (Rluc8). Rab5a-Venus (early endosome), Rab7a-Venus (late endosomes), Rab11-Venus (recycling endosome), βArr1-YFP, and βArr2-YFP were from N. Lambert (Augusta University Medical College of Georgia, GA).

For nbBRET studies, MrGPRX1-NP was cloned using Gibson Assembly with cDNA encoding human MrGRPX1 with an N-terminal POMC signal sequence and Flag tag in a pcDNA5/FRT vector. The 13 amino acid natural peptide fragment of nanoluciferase (GVTGWRLCERILA, NP) was insert to the C-terminus of MrGPRX1 via a flexible linker (LRPLGSSGGG). Localization markers CAAX (plasma membrane) or FYVE (endosome) tagged on the N-terminus with HA, a short linker (GGSG) and the LgBiT tag were donated by the Alex Thompson lab (New York University). Venus-miniGα_*q*_, and Venus-miniGα_*s*_ were from N. Lambert.

### Cell culture

Human Embryonic Kidney 293 (Thermofisher) were culture in Dulbecco’s modified Eagle’s medium (DMEM) supplemented with 10% normal fetal bovine serum (FBS) and 100 U/mL penicillin-streptomycin at 37 °C and 5% CO_2_. Human Embryonic Kidney 293 cell tetracycline-inducible system (T-Rex™293, Thermofisher) expressing TRPV1 (HEK-TRPV1) were culture in DMEM containing 10% tetracycline free FBS, 100 U/mL penicillin-streptomycin, 100 μg/mL Hygromycin B Gold (InvivoGen), and 5 μg/mL Blasticidin (InvivoGen) at 37 °C and 5% CO_2_. The expression of TRPV1 was induced overnight with 0.1 μg/mL tetracycline.

### Transfection of cell lines

HEK293-Flp-In cells were transfected using polyethylenimine (PEI, Polysciences; 1:6 DNA:PEI) diluted in a 150 mM NaCl solution.

### MrGPRX1 internalization

HEK293 cells were transfected with MrGPRX1-mApple with or without Dynamin K44A and were plated on PDL coated 12 mm cover slips in a 24 well plate. Following transfection (48 h), cells were challenged with vehicle or BAM8-22 (1 μM) for 15- or 30-min at 37 °C. Cells were washed with PBS and fixed with PFA (4% paraformaldehyde, in PBS, 20 min, on ice). Cells were blocked in 5% normal horse serum (NHS), 0.1% Saponin in PBS (1 h, RT), and incubated with rabbit anti-EEA1 (1:1,000, Invitrogen) overnight, 4°C. Cells were washed and incubated with anti-rabbit Alexa Fluor^®^ 488 (1:1,000, 1 h, RT; Invitrogen). Cover slips were washed 3X with PBS and mounted on glass slides with ProLong^®^ Diamont Antifade (Thermo Fisher). Sections were imaged using a Leica SP8 confocal microscope with HCX PL APO 63x (NA 1.40) oil objectives (Leica-Microsystems). Mander’s colocalization coefficients were quantified using the Just Another Colocalization Plugin (JACoP) in ImageJ using Otsu thresholding to determine signal to noise (Bolte and Cordelières, 2006). 6 independent experiments were performed. For each independent experiment, at least 3 technical replicates were processed, and a minimum of 5 cells were quantified per technical replicate. To ensure statistical rigor, the measurements were averaged within each independent experiment. Images were processed using ImageJ and figures were made in Adobe Illustrator.

### BRET transfections

For BRET assays of MrGPRX1 translocation to different organelles, HEK293 cells were transfected in 10 cm dishes with MrGPRX1-Rluc8 (1 μg) and Venus-CAAX (3 μg, plasma membrane), Venus-Rab5a (3 μg, early endosomes), Venus-Rab7a (3 μg, late endosomes), or Venus-Rab11a (3 μg, recycling endosomes). To disrupt Dynamin, HEK293 cells were co-transfected with Dynamin K44A (1 μg).

To characterize the recruitment of G proteins to the plasma membrane or to endosomes, HEK293 cells were transfected with human MrGPRX1-NP (2 μg) and HA-LgBiT-CAAX (2 μg) or HA-LgBiT-FYVE (2 μg), and either Venus-mGα_*q*_ (1 μg), Venus-mGα_*s*_ (1 μg), YFP-βArr1 (1 μg) or YFP-βArr2 (1 μg).

### BRET measurements

Following transfection (48 h), HEK293 cells were washed with HBSS containing 10 mM HEPES at pH 7.4. In some experiments, cells were pre-incubated in HBSS containing Pitstop 2 or Pitstop 2-negative control, a structural derivative of PS2 that lacks the ability to inhibit clathrin-mediated endocytosis (10 μM; Abcam), Dyngo4a (10 μM; Abcam), or vehicle for 30 min, 37°C. Prior to BRET measurements, substrate was added for eBRET (coelenterazine H, 2.5 μM, 10 min), or nbBRET (furimazine, 10 μM, 10 min). BRET was recorded for up to 25 min in a CLARIOstar Microplate reader (BMG Labtech, Cary NC) using BRET and nbBRET filters (donor filter: 460 ± 40 nm, acceptor filter: 540 ± 25 nm). Baseline was measured for 2–3 min and cells were then challenged with BAM8-22 or vehicle. The BRET signal was calculated as the ratio of the acceptor (YFP or Venus) emission over the donor (RLuc8, Nanoluc) emission. ΔBRET represents the BRET signal in the presence of agonist, minus the mean from 5 initial reads and baseline-corrected to vehicle. Area under the curve (AUC) was determined for each replicate.

### FRET biosensor assays

HEK293 cells were transfected with FRET biosensors (2 μg/10 cm dish), and MrGPRX1 (2 μg/10 cm dish) with or without a co-transfection of Dynamin K44A (1 μg/10 cm dish). After 24 h, cells were transferred to 96-well plates and serum-starved overnight for ERK activity assays. On the day of the assay, cells were washed and incubated in Hanks’ buffered saline solution (HBSS, +10 mM HEPES, pH 7.4) containing 0.1% BSA. In some experiments, cells were pre-incubated in HBSS containing Pitstop 2 or Pitstop 2-negative control, a structural derivative of PS2 that lacks the ability to inhibit clathrin-mediated endocytosis (10 μM; Abcam), Dyngo4a (10 μM; Abcam) or vehicle for 30 min, 37°C. FRET was measured at 60 s intervals (CLARIOstar, BMG Labtech). After 5 baseline reads, cells were stimulated with BAM8-22 (1 nM-10 μM). ΔFRET represents the ratio (YFP/CFP), minus the mean from 5 initial reads and baseline-corrected to vehicle. Area under the curve (AUC) was determined for each replicate.

### Luciferase transcriptional assays

HEK293 cells were transfected with luc2P vectors (2 μg/10 cm dish), and MrGPRX1 (2 μg/10 cm dish) with or without a co-transfection of Dynamin K44A (1 μg/10 cm dish). After 24 h, cells were transferred to 96-well plates and serum-starved overnight. On the day of the assay, cells were treated with vehicle or BAM8-22 (1 μM). After 4 h (37 °C, 5% CO_2_), cells were incubated with luciferin and lysis buffer for 5 min to enable the substrate reaction. Relative luminescence units (RLU) were measured (CLARIOstar, BMG Labtech).

### Calcium (Ca^2+^) assays in HEK293 cells

HEK293 cells expressing MrGPRX1, TRPV1, or MrGPRX1-TRPV1 were seeded onto poly-D-lysine coated 96-well clear plates (30,000 cells/well) and cultured for 24 h. Cells were loaded with Fura2-AM ester (1 μM) in Calcium Buffer (150 mM NaCl, 2.6 mM KCl, 1.18 mM MgCl2, 2.2 mM CaCl2, 10 mM Glucose, 10 mM HEPES, and 0.5% BSA)c supplemented with probenecid (0.04 mM) and pluronic acid (0.5 μM) for 60 min at 37° C. Fluorescence was measured at 340/380nm excitation and 510 nm emission wavelengths using a FlexStationIII plate reader. Baseline measurements were recorded for 45 s prior to agonist addition. Changes in (iCa2+) were monitored following a ratio of F340/F380, and data was normalized using the average of the baseline measurements. Cells were incubated for 30–60 min with Go 6983 inhibitor prior to agonist administration. For the sensitization of TRPV1 assay, BAM 8-22 was incubated 5 min prior Capsaicin addition.

### Animals

All animal experiments were adhered to the guidelines recommended by the National Institute of Health, the International Association for the study of Pain, the National Centre for the Replacement, Refinement and Reduction of Animals in Research (ARRIVE) guidelines (Kilkenny et al., 2010) and were conducted per the Guide for the Care and Use of Laboratory Animals. This study was approved by the Animal Ethics of The New York University Institutional Animal Care and Use Committee. Male and female C57BL/6J mice (#00064 Jax^®^, wild-type; RRID:IMSR_JAX:000664) were maintained in a temperature-controlled (22°C) environment with a 12 h light/dark cycle and access to food and water *ad libitum*. Mice were randomly assigned to experimental groups; group size was based on previous similar studies. Investigators were blind to treatments.

### Collection of mouse tissue

Mice were anesthetized (5% isoflurane) and perfused through the ascending aorta with PBS and then 4% paraformaldehyde in PBS. DRG (L4-L5) were removed, fixed in 4% paraformaldehyde in PBS (1 h, 4°C), cryoprotected in 30% sucrose (24 h, 4°C), and embedded in Optimal Cutting Temperature compound (Tissue Tek). Frozen sections (10–12 μm) were mounted, dried (15 min) and stored (−20°C).

### RNAScope^®^ *in situ* hybridization

mRNA transcripts were localized in mouse tissues using a RNAScope™ Multiplex Fluorescent Reagent v2 Assay Kit (Advanced Cell Diagnostics Inc.) using the protocol for fresh-frozen tissue as recommended by the manufacturer, except for omission of the initial on-slide fixation step with mouse tissue. Probes to mouse (Mm) Mm-mrgprx1, and Mm-trpv1 were used. To detect all neurons in mouse tissues, hybridized slides were blocked and incubated with guinea pig anti-NeuN antibody (1:500, EMD Millipore) overnight, 4°C. Slides were washed and incubated with goat anti-guinea pig Alexa Fluor^®^ 647 (1:1,000) 1 h, RT. Slides were washed, incubated with DAPI and mounted. Sections were imaged using a Leica SP8 confocal microscope with HCX PL APO 40x (NA 1.30) oil objective. The percentage of hybridized positive neurons were quantified and normalized by the total number of neurons.

### Mouse DRG cultures

C57BL/6 mice (∼4 weeks) were sacrificed following the guidelines of the American Veterinary Medical Association for the euthanasia of animals. Briefly mice were anesthetized via isoflurane (O_2_ 1.0 L/min + 4% isoflurane) and sacrificed via decapitation. Thoracic and lumbar DRG were excised and dissociated with papain (60 U/mL) in Hank’s balanced salt solution (HBSS) for 30 min at 37°C, then in collagenase (1 mg/mL) in HBSS for another 30 min at 37°C under gentle agitation. They were triturated with 1 ml pipette and passed through a 70-μm filter. Dissociated cells were pelleted and resuspended in DMEM containing 10% FBS and 1% penicillin-streptomycin. Neurons were plated on glass coverslips or on 35 mm glass bottom dishes (MatTek, Ashland MA) pretreated with poly-D-lysine (0.1 mg/mL). For AAV NucEKAR transduction, 1 μL of AAV virus was added to each coverslip or dish. Neurons were used within 72 h. The AAV-NucEKAR particles were designed by and purchased from GeneCopoeia using an AV13 serotype with a synapsin promoter to limit expression to neurons.

### Calcium (Ca^2+^) imaging in mouse DRG neurons

Mouse DRG neurons were seeded onto poly-D-lysine coated 35 mm glass bottom dishes (MatTek, Ashland MA) and cultured for 24 h. Changes in agonist-induced calcium influx were determined by loading the neurons with Fura2-AM ester (1 μM) in Calcium Buffer (150 mM NaCl, 2.6 mM KCl, 1.18 mM MgCl2, 2.2 mM CaCl_2_, 10 mM Glucose, 10 mM HEPES, and 0.5% BSA) supplemented with probenecid (0.04 mM) and pluronic acid (0.5 μM) for 60 min at 37 °C. Fluorescence was measured at 340/380 nm excitation and 530 nm emission wavelengths using Leica DMi8 Widefield microscope. Images were taken every ∼10 s during the time course of the experiment to minimize photobleaching and phototoxicity and to provide acceptable image quality. Changes in (iCa^2+^) were monitored following a ratio of F340/F380, calculated after subtracting the background from both channels. Baseline measurements were recorded for 60 s prior to agonist addition, and data was normalized using the average of these baseline measurements. Cells were incubated for 30–60 min with Go 6983, U0126, Pitstop 2, or Dyngo4a inhibitors prior to agonist administration. Cells were incubated overnight with ERK inhibitor FR 180204 prior to agonist administration. For the TRPV1 sensitization analysis, BAM8-22 was incubated 10 min prior to capsaicin addition, and cells that responded to BAM8-22 stimulus (MrGPRX1-positive) were analyzed.

### Behavioral test

Dyngo4A, U0126 (ERK inhibitor), GF109203X (PKC inhibitor) and SB705498 (TRPV1 antagonist) were dissolved in dimethyl sulfoxide (DMSO) into a stock solution and diluted in PBS prior to use. Dyngo4A (30 μM in 10 μL PBS), U0126 (0.5 μg in 5 μL PBS), GF109203X (0.25 μg in 5 μL PBS) or SB705498 (0.05 μg/5 μL) were administered intrathecally with a 30-gauge needle at the cervical level to anesthetized mice, 30 min prior to pruritogen injection. Control mice received an intrathecal injection of the same volume of 1% DMSO in PBS. Each mouse received a single intrathecal injection of either vehicle or the different inhibitors tested. After intrathecal injection, 10 μL of 10 μM BAM8-22 in saline was injected intradermally into the shaved nape of each mouse. Animals showing any signs of motor impairment post-injection were excluded from the study.

Immediately following the BAM8-22 injection, itch was assessed using an automatic and unbiased behavioral spectrometer (Behavior Sequencer, Behavioral Instruments, NJ; BiObserve, DE) (Brodkin et al., 2014; Castro et al., 2019; Latorre et al., 2022). The spectrometer comprised a 40-cm^2^ arena with a CCD camera mounted in the center of the ceiling and a door aperture in the front area of the arena. Movement was assessed by a floor mounted vibration sensor and 32 wall mounted infrared transmitter and receiver pairs. Mice were individually placed in the center of the behavioral spectrometer, and their behavior was recorded for 30 min and analyzed using a combination of video tracking analysis (Viewer3, BiObserve, Sankt Augustin, Germany) and vibration analysis. Total distance traveled in the open field (track length; m), time still (minutes), and time engaged in grooming (minutes), were recorded and analyzed as described. Scratching behavior was analyzed by the experimenter offline after the record. Each scratching bout was defined as rapid brushing of the back nape by the hind paw.

### Data analysis

Data presented as mean ± SD, unless noted otherwise. The data were first tested for a Gaussian distribution using Shapiro-Wilk or D’Agostino–Pearson test, depending on the sample size (GraphPad Prism 10 software). The statistical significance of differences between means was calculated using a Student’s *t*-test for two-group comparisons, and parametric analysis of variance (ANOVA) followed by a Dunnett’s or Tukey’s *post-hoc* test for multiple comparisons using GraphPad Prism 10 software. *Post-hoc* tests were run only if F achieved *P* < 0.05 and there was no significant variance inhomogeneity. *P* < 0.05 was considered significant at the 95% confidence level. All data were plotted in GraphPad Prism 10 software.

## Data Availability

The raw data supporting the conclusions of this article will be made available by the authors, without undue reservation.
