# Supplementary material for: Endosomal MrGPRX1 signaling sensitizes TRPV1 to enhance itch
Source: Front Mol Neurosci. 2026 Jul 1;19:1880762. doi: 10.3389/fnmol.2026.1880762 (PMC13368649; doi:10.3389/fnmol.2026.1880762)
Supplement: Supplementary file 1 [file Data_Sheet_1.DOCX]

**
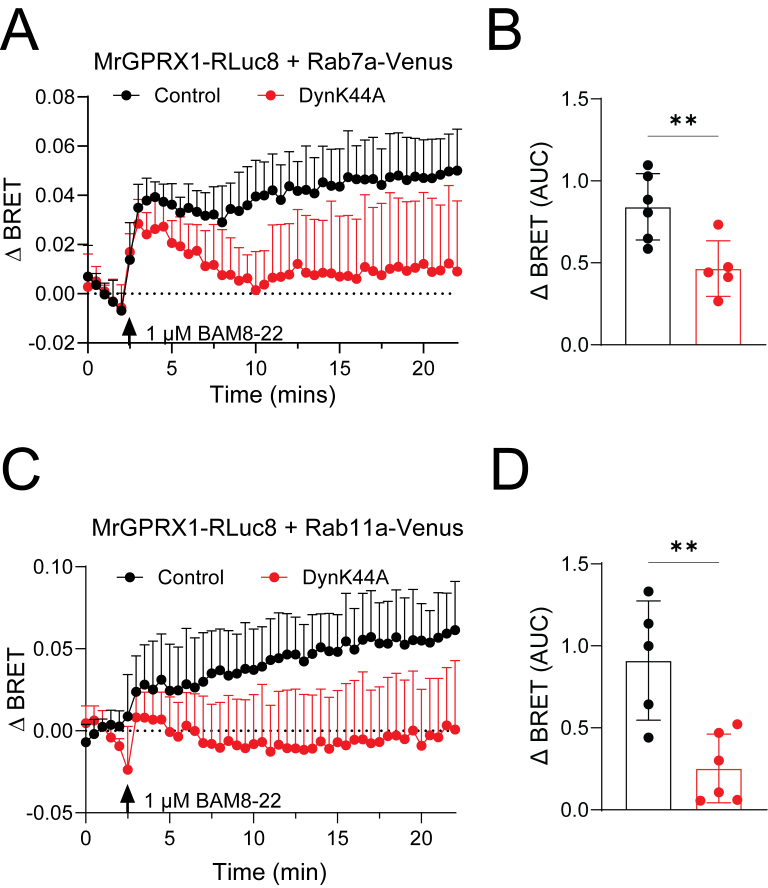
**

**Supplementary Figure 1. MrGPRX1 traffic can be blocked by dynamin inhibition.** BAM8-22-induced (1 µM) trafficking of MrGPRX1-Rluc8 to late endosomes (A) (Rab7a-Venus), and to (C) recycling endosomes (Rab11a-Venus) in HEK293 cells is blocked by DynK44A expression. Area under the curve (AUC) represents quantification of change in BRET over time. n≥5, ** P≤ 0.01. Unpaired t-test.

**
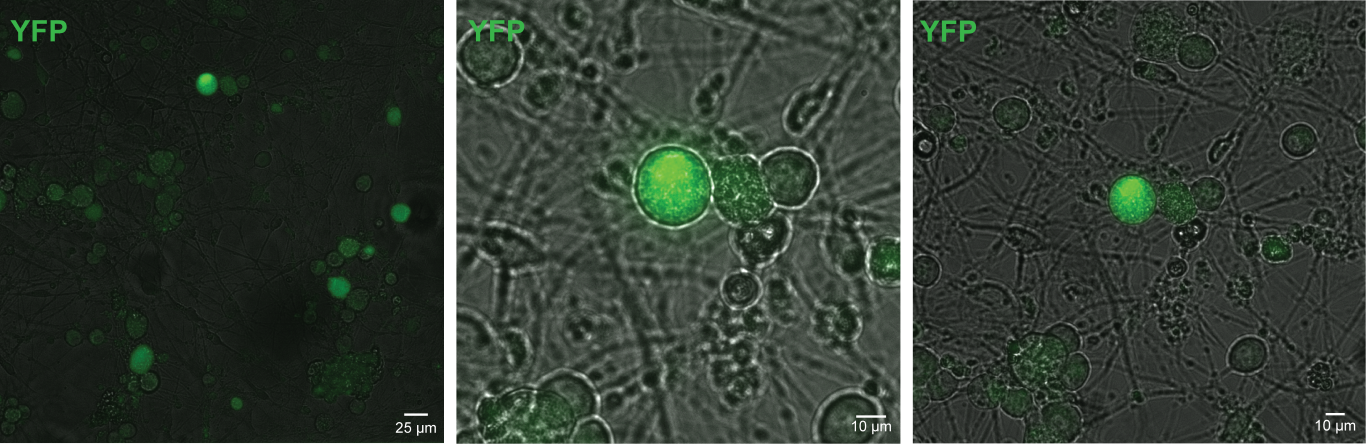
**

**Supplementary Figure 2. NucEKAR FRET biosensor expression in mouse dorsal root ganglion (DRG) neurons.** Representative image of mouse DRG neurons in culture transduced with adeno-associated virus (AAV-NucEKAR) nuclear ERK FRET biosensor. Scale, 25 µm, and 10 µm.

**
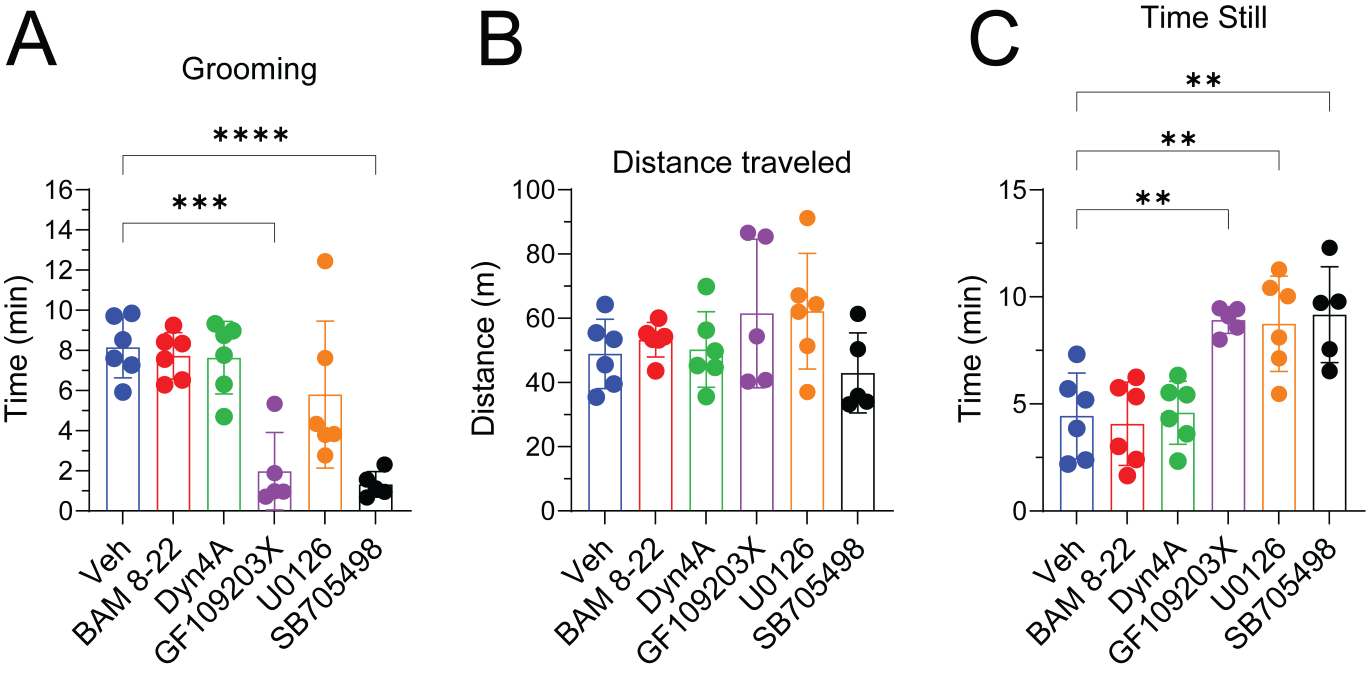
**

**Supplementary Figure 3. Effect of endocytic, MEK and PKC inhibitors in non-evoked behaviors.** Non-evoked behaviors (A) grooming, (B) distance traveled, and (C) time still of vehicle or BAM8-22 intradermal injection 30 min after intrathecal administration of endocytic inhibitor Dyngo4A, MEK inhibitor U0126, PKC inhibitor GF109203X or the TRPV1 antagonist SB705498. N≥5 mice/group, ** P≤ 0.01, *** P≤ 0.001, ****P≤0.0001. One-Way ANOVA, Tukey’s multiple comparisons.

**Supplementary Table 1** Catalog number and RRID

| **Resource Type** | **Resource Name** | **Manufacturer/Source** | **Catalog/Accession Number** | **RRID** |
| --- | --- | --- | --- | --- |
| **Cell line** | HEK293 | Invitrogen | R75007 | CVCL_U421 |
|  | HEK293 Flp-In T-Rex | Invitrogen | R78007 | CVCL_U427 |
| **Plasmids** | Cytoplasmic EKAR | Addgene | 18679 | Addgene_18679 |
|  | Nuclear EKAR | Addgene | 18681 | Addgene_18681 |
|  | Cytoplasmic CKAR | Addgene | 14870 | Addgene_14870 |
|  | DynaminK44A | Addgene | 34683 | Addgene_34683 |
|  | pGL4.33[luc2P/SRE/Hygro] | Promega | E1430 | Promega_E1340 |
|  | pGL4․30[luc2P⁄NFAT-RE⁄Hygro] | Promega | E8481 | Promega_E8481 |
|  | pGL4․29 [luc2P⁄CRE⁄Hygro] | Promega | E8471 | Promega_E8471 |
|  | DMEM | Gibco | 10566016 | Thermo_Fisher_  10566016 |
| **Cell Culture** | FBS | Gibco | 16000044 | Thermo_Fisher_  16000044 |
|  | Tet-Free FBS | Gibco | 16000044 | Thermo_Fisher_  16000044 |
|  | Penicillin-Streptomycin | Gibco | 15140122 | Thermo_Fisher_  15140122 |
|  | Hygromycin B Gold | InvivoGen | Ant-hg-1 | InvivoGen_ant-hg-1 |
|  | Blasticidin | InvivoGen | Ant-bl-1 | InvivoGen_ant-bl-1 |
|  | Polyethylenimine (PEI) | Polysciences | 06088 | Polysciences_06088 |
|  | Mm-mrgprx1 | Advanced Cell Diagnostics | 488771 | ACDBio_488771 |
| **Probes** | Mm-trpv1-C3 | Advanced Cell Diagnostics | 313331-C3 | ACDBio_313331-C3 |
|  | guinea pig anti-NeuN | EMD Millipore | ABN90 | AB_11205592 |
| **Antibodies** | Rabbit Anti-EEA1 | Invitrogen | PA1-063A | AB_2096819 |
|  | goat anti-guinea pig Alexa Fluor® 647 | Thermofisher | A-21450 | AB_2535867 |
|  | BAM8-22 | Tocris | 1763 | Tocris_1763 |
| **Reagents** | Capsaicin | Sigma-Aldrich | M2028 | Sigma-Aldrich_M2028 |
|  | GF 109203X | Tocris | 0741 | Tocris_0741 |
|  | FR 180204 | Tocris | 3706 | Tocris_3706 |
|  | U0126 | Tocris | 1144 | Tocris_1144 |
|  | Gö 6983 | Tocris | 2285 | Tocris_2285 |
|  | SB705498 | Tocris | 5249 | Tocris_5249 |
|  | Coelenterazine h | NanoLight Tech | 301 | NanoLight_Technology  _301 |
|  | Nano-Glo Substrate | Promega | N1110 | Promega_N1110 |
|  | Fura-2AM | Cayman Chemical | 20421 | Cayman_Chemical  _20421 |
|  | Dyngo4a | Abcam | Ab120689 | Abcam_ab120689 |
|  | PitStop2 | Abcam | Ab120687 | Abcam_ab120687 |
